# Research Notes: Evaluation of recombinant Newcastle disease virus expressing gB protein of infectious laryngotracheitis virus as bivalent in ovo vaccine

**DOI:** 10.1016/j.psj.2024.104397

**Published:** 2024-10-10

**Authors:** Liren Jiang, Helong Feng, Zhe Zeng, Zichen Wang, Gaofeng Zhang, Yu Shang, Hongcai Wang, Shixin Wang, Lun Yao, Li Li, Xiaoyu Suo, Qingping Luo, Guoyuan Wen

**Affiliations:** ⁎ Institute of Animal Husbandry and Veterinary Sciences, Hubei Academy of Agricultural Sciences, Wuhan, China; † Hubei Provincial Key Laboratory of Animal Pathogenic Microbiology, Wuhan, China; ‡ Key Laboratory of Prevention and Control Agents for Animal Bacteriosis (Ministry of Agriculture), Wuhan, China; § Hubei Hongshan Laboratory, Wuhan, China; # Shannan Tibetan Chicken Industry Research Institute, Tibet Shannan 856000, China

**Keywords:** Newcastle disease virus, in ovo vaccine, glycoprotein gB, infectious laryngotracheitis virus

## Abstract

Infectious laryngotracheitis (**ILT**) and Newcastle disease (**ND**) are 2 highly infectious avian respiratory diseases that have caused significant economic losses in the poultry industry worldwide. In ovo vaccination is administered during the late stage of incubation and is an attractive immunization method for poultry industry. However, most of the avian live vaccine strains that are safe for use after hatching are highly pathogenic to chicken embryos and therefore unsafe for in ovo vaccination. Previously, a recombinant Newcastle disease virus (**NDV**) strain, rTS-gB, expressing the gB protein of Infectious laryngotracheitis virus (**ILTV**), was demonstrated to be safe and immunogenic as a bivalent vaccine for hatched birds. In this study, we evaluated the safety and protective efficacy of rTS-gB as an in ovo vaccine. This vaccine strain was found to be safe for in ovo vaccination, with a hatchability and survival rate of 93.3% in chickens vaccinated in ovo with rTS-gB. In ovo vaccination with rTS-gB induced effective ILTV- and NDV-specific antibody responses in birds and conferred complete protection against virulent NDV and ILTV challenges. Furthermore, shedding of the challenged ILTV in cloacal and tracheal samples from in ovo vaccinated chickens was greatly reduced. These results indicate that the rNDV strain rTS-gB is a safe and highly immunogenic in ovo vaccine candidate against both ND and ILT.

## INTRODUCTION

Infectious laryngotracheitis (**ILT**) is a highly contagious acute respiratory tract disease caused with infectious laryngotracheitis virus (**ILTV**), the disease is characterized by respiratory distress, swollen orbital sinuses and mortality reaching up to 70% (Gowthaman et al., 2020). Vaccination is a common strategy for ILT control, chicken embryo origin (**CEO**) and tissue culture origin (**TCO**) attenuated vaccines have been widely used worldwide. However, the 2 vaccines cause adverse reactions due to viral swift replication, can regain virulence and become the source of outbreaks, limiting their use in poultry flocks (García and Zavala, 2019). The recombinant vaccines, such as the fowl pox virus (**FPV**) and turkey herpesvirus (**HVT**) vectors encoding ILTV antigens are considered to be safer alternatives, but these vaccines are hard to stop complete viral shedding, and induce only partial protection (Johnson et al., 2010). Therefore, there is a significant need to develop safer and more efficacious ILT vaccines.

Newcastle disease (**ND**) is one of the most serious avian diseases, and caused with Newcastle disease virus (**NDV**). The avirulent NDV strains replicates only in the respiratory and digestive tracts of poultry, and have been used as live vaccines and vaccine vector worldwide. Compared with other viral vaccine vectors, NDV can induce robust mucosal immunity except for humoral and cellular immunity (Duan et al., 2015). The various avian disease recombinant vaccines using NDV as vector, such as H9N2 subtype avian influenza, infectious bronchitis, have been proven to be highly effective in protecting chickens (Shao et al., 2022; Shirvani et al., 2018). The conventional delivery methods of ND and ILT live attenuated vaccines, including spraying, in drinking water, and in feed, can result in inconsistent vaccine delivery and consequent poor vaccine efficacy (Sokale et al., 2017).

In ovo vaccination, administered during the late incubation stage, is an appealing immunization method for the poultry industry due to its ability to reduce human error, ensure uniform vaccine delivery, and close the susceptibility window (Peebles, 2018). The NDV live vaccines and recombinant ILT vaccine (such as rHVT-LT and rFPV-LT) had been approved for in ovo delivery (Dilaveris et al., 2007; García and Zavala, 2019). The most of NDV vaccines could kill embryos used as in ovo vaccines, and not yet commercially available (Feng et al., 2023). It was proved that the rFPV-LT vaccine could cause granulomatous bronchopneumonia lesions in hatched chickens. Although the rHVT-LT vaccine could reduce clinical sings of ILT, but they were hard to limit the challenge virus replication in chickens (García and Zavala, 2019).

Previously, we reported that NDV strain TS09-C was a safe in ovo vaccine, and a NDV recombinant expressing the ILTV glycoprotein B (**rTS-gB**) was developed as bivalent vaccine based on TS09-C, which was highly efficient candidate vaccine to immunize 4-week-old SPF chickens against ILTV(Zeng et al., 2023). In this study, the safety, immunogenicity and protective effect of rTS-gB strain was evaluated as in ovo vaccine.

## MATERIALS AND METHODS

### Ethics Statement

All the animal experiments were approved (Permit number 05/2023) and supervised by the Institutional Animal Care and Use Committee of the Hubei Academy of Agriculture Sciences, Wuhan, China.

### Eggs, Cells, and Viruses

One hundred un-incubated Specific-pathogen-free (**SPF**) Leghorn chicken embryos were purchased from Merial-Vital, Beijing, China. The SPF chicken embryos were incubated for 22 d in an automatic incubator (Yunxiang Incubation Equipment Factory, Shandong, China). Baby Syrian hamster kidney (**BHK-21**) cells were obtained from the China Center for Type Culture Collection (Wuhan, China). NDV strains TS09-C, LaSota, F48E9 and ILTV strain WG were obtained from Pathogen Repository Bank at Hubei Academy of Agriculture Sciences. The GenBank accession numbers of strains were JX110635, JF950510, MG456905 and JX458823, respectively. Recombinant NDV strain rTS-gB were previously generated based on TS09-C backbone (Zeng et al., 2023).

### In ovo Vaccination

The method of in ovo vaccination has been described previously (Feng et al., 2023). Briefly, 90 SPF chicken embryos (18-day-old) were divided randomly into 3 groups, and cleaned with 75% alcohol, a 1-mm hole was knocked out on the top of the chicken embryos. Chicken embryos from 3 groups were inoculated with TS09-C strain, rTS-gB strain at a dose of 10^3.0^ 50% egg infectious dose (**EID_50_**)/egg, or PBS, via the amniotic route with a 38 mm 23G needle at a depth of 1 inch. The vaccinated eggs were sealed and hatched.

### Serological Testing

Blood samples were collected from 10 birds of each group to assess NDV- and ILTV-specific antibody titers at 7, 14, 21, 28 d posthatching (**dph**). Four units of NDV strain LaSota were used as antigens to detect hemagglutination inhibition (**HI**) antibody titers. ILTV-specific antibody titers were detected with the previous established laboratory-developed enzyme-linked immunosorbent assay (**ELISA**) (Zeng et al., 2023). Briefly, the purified gB protein was used for coating in 96-well plates at 4°C overnight, and then serum samples were added after blocking. The HRP-labeled anti-chicken secondary antibody was added to each well. TMB chromogenic solution was added after washing 3 times, and the absorbance value was measured at OD_630nm_.

### Challenge Test, Survival Rate and Clinical Signs Statistics

Challenge experiments were performed at 28 dph. For NDV, 13 birds from each group were infected with the virulent NDV strain F48E9 at a dose of 10^5^ 50% embryo lethal doses (**ELD_50_**)/bird via intramuscular route, and the mortality of chickens was counted daily until 14 d postchallenge (**dpc**). For ILTV, 13 birds from each group were infected with WG strain at a dose of 2 × 10^4^ EID_50_ per bird in a total volume of 100 μL. The challenge was administered via intratracheal and intraocular routes, with 50 μL used for each route, respectively. After the challenge, chickens from each group were placed separately in isolators (Suhang Technology Equipment Co., Ltd, Suzhou, China). The clinical signs of chickens were monitored every 12 h until 14 dpc. The severity of respiratory signs, conjunctivitis and depression was scored on a scale of 0-3: healthy = 0 (No clinical signs were observed), mild = 1 (depressed, mild dyspnea and open mouth breathing, swollen and/or partial closure of the eyes), severe = 2 (severely depressed, gasping with an extended neck, complete closure of the eyes), and death = 3.

### Tissue Viral Load After NDV Challenge

The lungs, tracheas and duodenums were collected to detect viral load at 3 dpc. All tissues were weighed, 1 mL volume of sterile PBS was added and ground, centrifuged at 10,000 rpm/min for 3 min. When BHK-21 cells were reached 80% confluence, 20 μL of the supernatant was added to 180 μL serum-free Dulbecco's modified Eagle's medium (**DMEM**) for serial dilution and added in 96-well cell culture plates. The virus titer of these tissues was detected by indirect immunofluorescence at 72 h postinfection (**hpi**). Briefly, the cells grown in a 96-well plate were fixed with 4% paraformaldehyde. After completion of fixation, cells were blocked in PBS containing 1% bovine serum albumin (**BSA**) and incubated with primary antibody (chicken anti-NDV antibodies) for 2 h and then with secondary antibody (fluorescein isothiocyanate [**FITC**]-labelled goat anti-chicken IgG) for 1 h. The number of positive wells was counted under a fluorescence microscope, and the 50% tissue culture infective dose (**TCID_50_**) value was calculated using the Reed-Muench method.

### Histopathology Tests, Tissue Viral Load, Virus Shedding After ILTV Challenge

Larynx and trachea were collected to assess histopathology and viral load of these tissues at 3 dpc (n = 3). Trachea and cloacal swabs were collected to detect viral shedding at 1, 3, 5, and 7 dpc (n = 10). Parts of the larynx and trachea tissues collected were fixed in 4% paraformaldehyde, paraffin embedded, sectioned and stained with hematoxylin–eosin, and analyzed microscopically. Virus titer of larynx and trachea tissues and trachea and cloacal swabs were detected by real-time fluorescence quantitative PCR. Standard curves of ILTV gC gene were previously established (Zeng et al., 2023). The final volume of the reaction was as follows: 5 μL of 2 × SYBR qPCR Master mix, primers at a final concentration of 0.125 µM, and 1 μL of DNA temple. The reaction cycling profile was as follows: After predenaturation at 95°C for 5 min, a 2-step PCR was performed, with 40 cycles of denaturation at 95°C for 10 s and annealing/extension at 60°C for 30 s. The cycle threshold (**CT**) value of the sample was transformed into the copy number by the standard curve. The sample with a copy number was considered positive, and the sample without a copy number was considered negative.

### Statistical Analysis

Antibody titers, viral loads, and viral shedding were analyzed by one-way analysis of variance (**ANOVA**) based on GraphPad Prism (Prism version 8.0.2) software, coupled with Tukey's test was used for multiple comparisons. Differences were considered significant at *P* values <0.05 (*, *P* < 0.05; **, *P* < 0.01; ***, *P* < 0.001).

## RESULTS AND DISCUSSION

### Safety and Immunogenicity of rTS-gB as an in ovo Vaccine

**Figure 1:**
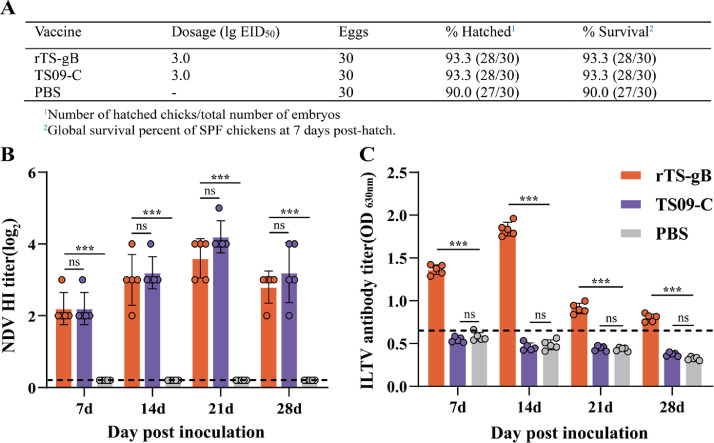
Safety and immunogenicity of rTS-gB after in ovo vaccination. (A) Hatchability and survival rate of chickens in ovo vaccinated with rTS-gB, TS09-C, or PBS. (B) and (C) The NDV-specific antibody and ILTV-specific antibody. The antibody titer was respectively detected by HI assay and ELISA. Each bar indicates the mean ± one standard deviation, statistics-based differences are marked by asterisks (*, *P* <0.05; **, *P* < 0.01; ***, *P* < 0.001).

To assessed the safety and immunogenicity of rTS-gB as an in ovo vaccine, 18-d chicken embryos were inoculated with rTS-gB, TS09-C, or PBS, respectively. The hatchability and survival rate of rTS-gB group were both 93.3%, similar to those of PBS group and TS09-C group (Figure 1A). The NDV HI and ILTV-specific antibody levels was assessed by HI and ELISA assays, respectively. As shown in Figure 1B, the HI titer of rTS-gB group was 3.6 ± 0.5 log_2_ at 21 dph, and no significant difference with TS09-C group (*P* > 0.05). The rTS-gB strain induced high level ILTV-specific antibody responses in hatched chickens, and the antibody titers were significantly higher than TS09-C and PBS group at 7, 14, 21, 28 dph (*P* < 0.01) (Figure 1C). These results demonstrated that rTS-gB strain was safety and immunogenicity as in ovo vaccine.

The commercial NDV vaccines such as LaSota, V4 strain were unsafe as in ovo vaccines, and the survival rate of chicken embryos vaccinated in ovo with LaSota and V4 strain were respectively 0% and 13.3% (Feng et al., 2023). For ILTV, the attenuated strain was obtained by deleting the virulence factor of ILTV, and tried to use as an in ovo vaccine. When the virulence factor glycoprotein G deficient strain of ILTV (ΔgG–ILTV strain) was used as in ovo vaccine, the hatchability of 10^2^ pfu dose group was 83.3%, however, the hatchability of 10^4^ pfu dose group was 58.3%, and indicated that the strain still had virulence to chicken embryos (Legione et al., 2012). The attenuated gJ-deleted ILTV strain (NΔgJ strain) was evaluated safety and efficacy for in ovo, the hatchability of chicken embryos inoculated with different doses of NΔgJ strain is 91 to 93%, but the mortality of hatching birds was from 2 to 28.6% (Mashchenko et al., 2013).

### Protective Efficacy Against Virulent NDV and ILTV Challenge

**Figure 2:**
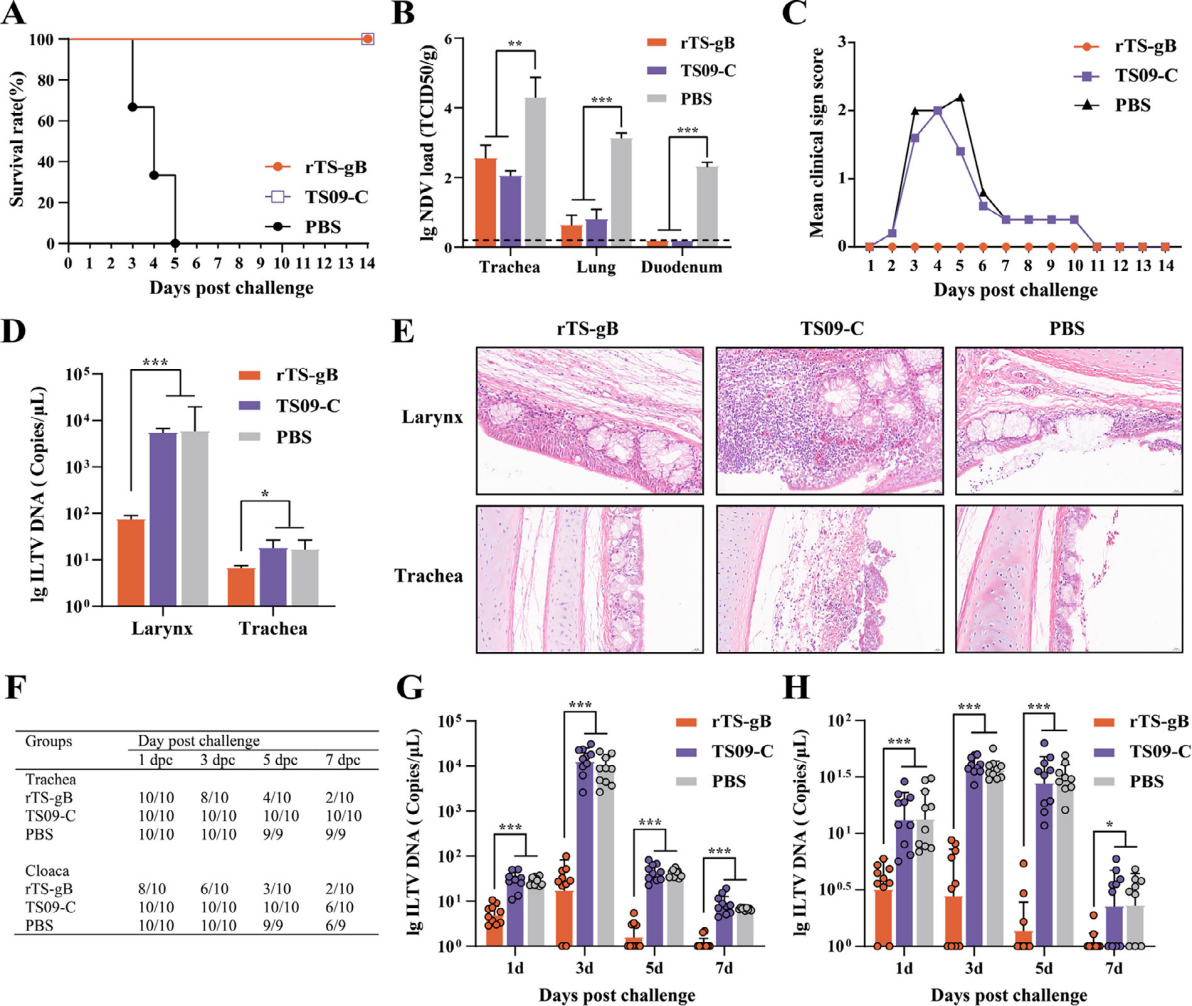
Protective efficacy of rTS-gB against NDV and ILTV challenge. (A) and (B) Survival rate and tissues virus load of chickens after challenged with virulent NDV F48E9 strain. The trachea, lung, and duodenum of chickens were collected at 3 dpc, and the virus titers of each sample were determined in BHK-21 cells. (C) and (D) Clinical sign score and tissues virus load of chickens after challenged with virulent ILTV WG strain. The larynx and trachea of chickens were collected at 3 dpc, and the virus titers of each sample were determined by qPCR. (E) Histopathological analysis of larynx and trachea from chickens challenged with ILTV at 3 dpc. (F) Virus shedding rate in trachea and cloacal swabs after challenged with ILTV at 1, 3, 5, 7 dpc. (G) and (H) The titers of trachea and cloacal swabs. Each bar indicates the mean ± one standard deviation. Statistics-based differences are marked by asterisks (*, *P* <0.05; **, *P* < 0.01; ***, *P* < 0.001).

At 28 dph, the birds of each group were challenged with virulent NDV or ILTV. Firstly, we evaluated protective efficacy against virulent NDV. As shown Figure 2A, no clinical symptoms or deaths were observed in the rTS-gB and TS09-C group after challenged virulent NDV, and all birds of PBS group died at 5 dpc (Figure 2A). The viral load of the trachea, lung, duodenum of birds challenged with NDV was detected at 3 dpc. The virus titer of the trachea and lung in rTS-gB group and TS09-C group was significantly lower than PBS group (*P* < 0.01), and no significant difference was observed between rTS-gB and TS09-C vaccine group (*P* > 0.05). The virus titer of duodenum in PBS group was 2.3 ± 0.1 lg TCID_50_/g, and virus was not detected in rTS-gB and TS09-C group (Figure 2B). These results demonstrate that rTS-gB vaccine provides effective protection and significantly reduces viral replication in respiratory and digestive tract tissues after virulent NDV challenged.

Secondly, we evaluated protective efficacy of rTS-gB against virulent ILTV. After challenged virulent ILTV, the typical clinical signs were observed in the birds of PBS and TS09-C group from 2 dpc, such as depression, conjunctivitis, or death, and showed the disease peak at 3 to 6 dpc. One bird in the PBS group dead at 5 dpc. In contrast, no clinical signs were observed in all birds of rTS-gB group during the observation period (Figure 2C). Lesions resulting from ILT are usually restricted to upper respiratory tract (Gowthaman et al., 2020). Therefore, we further evaluate the viral load and histopathological lesions of the upper respiratory tract tissues (larynx and trachea) after challenged. The high viral load of the larynx and trachea in TS09-C and PBS group was detected, and significantly higher than those in rTS-gB group (*P* < 0.05) (Figure 2D). As shown Figure 2E, moderate histopathological lesions were observed in larynx (hemorrhage, glandular necrosis, cilia desquamating) and trachea (submucosal edema, glandular necrosis, cilia desquamating) from birds of TS09-C and PBS group. In contrast, no histopathological lesions were observed in the larynx and trachea from birds of rTS-gB group. These results demonstrated that rTS-gB could significantly reduce clinical signs and viral replication in respiratory tract tissues, and provide good protection to against histopathological lesions caused with ILTV.

The reduction in virus shedding after challenge is an important criterion to evaluate the efficacy of the vaccine (Gowthaman et al., 2020; Zeng et al., 2023). The ILTV DNA copies of trachea and cloacal swabs was detected by relative qPCR. As shown Figure 2F, rTS-gB significantly reduced the shedding of challenged virus in trachea swabs from 5 dpc and cloacal swabs from 3 dpc. The conventional vaccines including live attenuated ILTV vaccines and recombinant vector ILTV vaccines frequently fail to reduce virus shedding after challenged with ILTV (Gowthaman et al., 2020). The rate of virus shedding of both trachea and cloacal swabs was only 20% (2/10) in rTS-gB group at 7 dpc. However, the rate of virus shedding of trachea swabs was both 100% in TS09-C group (10/10) and PBS group (9/9), and the rate of virus shedding of cloacal swabs was respectively 60% (6/10) in TS09-C group and 66.7% (6/9) in PBS group (Figure 2F). Furthermore, the virus titers of trachea and cloacal swabs rTS-gB group were both significantly lower than those in PBS and TS09-C group at 1, 3, 5, and 7 dpc (*P* < 0.05) (Figure 2, Figure 2). These results suggested that rTS-gB as in ovo vaccine could effectively reduce the shedding of challenged ILTV in chickens.

Approximately 30 yr ago, in ovo technology first became available for vaccination delivery in broiler hatcheries. In 1995, in ovo technology was used to immunize 55% of broiler embryos against Marek's disease in North America. In ovo mechanized injector can provide uniform and fast delivery of vaccines, up to 62,000 eggs per hour, and the automated machines is used in more than 90% of United States broiler production (Peebles, 2018). Overall, our results indicate that rTS-gB is a highly promising in ovo vaccine for the control of ND and ILT, and it offers new insights for the development of in ovo vaccines.

## DISCLOSURES

The authors declare no conflicts of interest.
